# Hypodermic Medication

**Published:** 1886-02

**Authors:** 


### Hypodermic Medication.

From Dr. Talfourd Jones' address before the British Medical Association, we learn that Dr. Alexander Wood, of Edinburgh, in 1885, published an account of his method of introducing liquor morphia into the system by subcutaneous injection, which was the first recommendation of the hypodermic method. It is an important point to know that the cumulative action of drugs is less when thus given than by any other way; elimination commences sooner and is sooner completed. Dr. Jones advises us to look carefully into the graduations on the piston-rod of the syringe, for he has found them more often wrong than right. While recommending the tablets for hypodermic use, Dr. J., recommends us to make our own solutions, *when we are ready to use them;* the majority of solutions (especially weak ones) do not keep well. The acetate of morphia he prefers to any other preparation of this salt. A *stock* bottle may be made by half-filling with water (not distilled), a bottle that holds *exactly* one ounce; put in 40 grains of acetate of morphia and *exactly* four minims of acetic acid; shake and fill the bottle with water. This solution will keep (if corked and kept in the dark) for six months. It should only be opened to fill the case bottle. It may become a little darker, but this is immaterial. A most important question arises in connection with the dose; as a general rule, we may say that, other things being equal, the dose of a drug must be apportioned according to the body weight of the patient. The hypodermic use of morphia should be avoided, if at all possible, with children, and when demanded, after having made due allowance for body weight, we should give no more than half the otherwise proportional dose. The initial dose should be from $\frac{1}{24}$ to $\frac{1}{4}$ of a grain. A solution of atropine will keep a little better if chloroform-water or camphor-water is used instead of plain water.

